# The Association between General Anesthesia and New Postoperative Uses of Sedative–Hypnotics: A Nationwide Matched Cohort Study

**DOI:** 10.3390/jcm11123360

**Published:** 2022-06-11

**Authors:** Chen-Yu Tai, Hsin-Yi Liu, Juan P. Cata, Ying-Xiu Dai, Mu-Hong Chen, Jui-Tai Chen, Tzeng-Ji Chen, Hsiang-Ling Wu, Yih-Giun Cherng, Chun-Cheng Li, Chien-Wun Wang, Ying-Hsuan Tai

**Affiliations:** 1 Department of Anesthesiology, Shuang Ho Hospital, Taipei Medical University, New Taipei City 23561, Taiwan; 18240@s.tmu.edu.tw (C.-Y.T.); 18384@s.tmu.edu.tw (H.-Y.L.); 19240@s.tmu.edu.tw (J.-T.C.); stainless@s.tmu.edu.tw (Y.-G.C.); 15193@s.tmu.edu.tw (C.-C.L.); 2 Department of Anesthesiology, School of Medicine, College of Medicine, Taipei Medical University, Taipei 11031, Taiwan; 3 Department of Anesthesiology and Perioperative Medicine, The University of Texas MD Anderson Cancer Center, 1515 Holcombe Blvd, Unit 409, Houston, TX 77030, USA; jcata@mdanderson.org; 4 Department of Dermatology, Taipei Veterans General Hospital, Taipei 11217, Taiwan; daiinxiu@gmail.com; 5 School of Medicine, National Yang Ming Chiao Tung University, Taipei 11221, Taiwan; kremer7119@gmail.com (M.-H.C.); tjchen@vghtpe.gov.tw (T.-J.C.); hlwu9@vghtpe.gov.tw (H.-L.W.); 6 Department of Psychiatry, Taipei Veterans General Hospital, Taipei 11217, Taiwan; 7 Department of Family Medicine, Taipei Veterans General Hospital, Taipei 11217, Taiwan; 8 Department of Family Medicine, Taipei Veterans General Hospital, Hsinchu Branch, Hsinchu 31064, Taiwan; 9 Department of Anesthesiology, Taipei Veterans General Hospital, Taipei 11217, Taiwan

**Keywords:** anxiolytic, benzodiazepine, risk factor, sleep disorder, sleep disturbance

## Abstract

Sedative–hypnotic misuse is associated with psychiatric diseases and overdose deaths. It remains uncertain whether types of anesthesia affect the occurrence of new postoperative uses of sedative–hypnotics (NPUSH). We used reimbursement claims data of Taiwan’s National Health Insurance and conducted propensity score matching to compare the risk of NPUSH between general and neuraxial anesthesia among surgical patients who had no prescription of oral sedative–hypnotics or diagnosis of sleep disorders within the 12 months before surgery. The primary outcome was NPUSH within 180 days after surgery. Multivariable logistic regression models were used to calculate the adjusted odds ratio (aOR) and 95% confidence interval (CI). A total of 92,222 patients were evaluated after matching. Among them, 15,016 (16.3%) had NPUSH, and 2183 (4.7%) were made a concomitant diagnosis of sleep disorders. General anesthesia was significantly associated both with NPUSH (aOR: 1.17, 95% CI: 1.13–1.22, *p* < 0.0001) and NPUSH with sleep disorders (aOR: 1.11, 95% CI: 1.02–1.21, *p* = 0.0212) compared with neuraxial anesthesia. General anesthesia was also linked to NPUSH that occurred 90–180 days after surgery (aOR: 1.12, 95% CI: 1.06–1.19, *p* = 0.0002). Other risk factors for NPUSH were older age, female, lower insurance premium, orthopedic surgery, specific coexisting diseases (e.g., anxiety disorder), concurrent medications (e.g., systemic steroids), postoperative complications, perioperative blood transfusions, and admission to an intensive care unit. Patients undergoing general anesthesia had an increased risk of NPUSH compared with neuraxial anesthesia. This finding may provide an implication in risk stratification and prevention for sedative–hypnotic dependence after surgery.

## 1. Introduction

Sedative–hypnotic misuse is a growing public health problem, affecting about 2–3% of the adult population worldwide [1,2]. Epidemiological study has shown that benzodiazepines and Z-drugs (i.e., zopiclone and zolpidem) were the third most commonly misused drugs in the United States in 2017 [1,2]. Sedative–hypnotic misuse is associated with psychiatric disorders, impaired quality of life, and overdose deaths [3,4]. However, the initial source of sedative–hypnotics among long-term users remains poorly understood.

Mounting evidence has shown that surgery, general anesthesia, opioids, and pain may contribute to the development of postoperative sleep disturbances by disrupting the sleep/wake cycle and changing sleep architecture [5,6,7,8,9,10,11,12,13]. Opioids used in general anesthesia can significantly reduce the time percentage of deep sleep and induce or exacerbate both central and obstructive sleep apnea [6,7,8]. An animal model demonstrated that sevoflurane inhalation induced rapid-eye-movement (REM) sleep deficits, delayed REM sleep recovery, and reduced latency to REM sleep [9]. In contrast, regional anesthesia reduces perioperative opioid consumption and alleviates postoperative pain, which may improve the sleep quality of surgical patients [7,10]. Nevertheless, some studies reported that sleep disturbances occur regardless of reduced opioid consumption and adequate pain relief among patients receiving neuraxial anesthesia [11,12,13].

Although both general and neuraxial anesthesia potentially relate to postoperative sleep disturbances, no study has compared the rates of postoperative sedative–hypnotic prescriptions between these two anesthetic techniques. Wright et al. recently reported that perioperative uses of benzodiazepines were associated with postoperative persistent uses of benzodiazepines, which may develop into long-term sedative–hypnotic misuse [14]. However, the perioperative influential factors for postoperative sedative–hypnotic uses are largely unknown.

We utilized Taiwan’s National Health Insurance (NHI) research database to conduct a nationwide population-based cohort study. There were two objectives in this study. First, we aimed to compare the risk of new postoperative uses of sedative–hypnotics (NPUSH) between general and neuraxial anesthesia in surgical patients. Second, we sought to evaluate the perioperative risk factors for NPUSH to identify potentially modifiable factors. This may provide important evidence in reducing postoperative sedative–hypnotic uses and preventing long-term misuse and its adverse effects among surgical patients. Based on the current evidence [5,6,7,8,9,10], we hypothesized that general anesthesia was associated with higher risks of NPUSH and new-onset sleep disorders compared with neuraxial anesthesia.

## 2. Material and Methods

### 2.1. Source of Data

This study obtained the approval from the Institutional Review Board of Taipei Medical University in Taiwan (TMU-JIRB-N202101005; data of approval on 7 January 2021). Written informed consent was waived by the Institutional Review Board. All methods of this study were performed in accordance with relevant guidelines and regulations [15]. Taiwan’s National Health Insurance program was launched in March 1995 and offered insurance to more than 99% of 23.5 million Taiwanese residents. The NHI research database contains comprehensive data of the insured beneficiaries, including demographic characteristics (e.g., date of birth and sex) and claims data (e.g., medical diagnoses, prescription drugs, interventional or diagnostic procedures, and medical expenditures). The NHI research data have been broadly used in epidemiological studies [16,17,18]. This study used three Longitudinal Health Insurance Databases (LHID2000, LHID2005, and LHID2010), which randomly sampled 1 million people from the original NHI research database in the years 2000, 2005, and 2010, respectively. The representativeness of LHIDs has been validated by Taiwan’s National Health Research Institutes [19].

### 2.2. Patient Selection

We used the medical claims of 3 million insured individuals to select patients who were aged ≥20 years and underwent their first surgery requiring general or neuraxial anesthesia from 1 January 2002 to 30 June 2013. We excluded surgeries that could only be performed with general anesthesia, surgeries with a length of hospital stay < 2 days, patients who were prescribed any oral sedative–hypnotics or had any diagnoses of sleep disorders within 12 months before the index surgery, and patients who died within 180 days after the index surgery. Oral sedative–hypnotics included benzodiazepine drugs (diazepam, chloradiazepoxide, lorazepam, bromazepam, alprazolam, medazepam, oxazepam, fludiazepam, oxazolam, nitrazepam, flunitrazepam, lorametazepam, estazolam, triazolam, brotizolam, midazolam, nimetazepam, flurazepam, and clonazepam) and non-benzodiazepine drugs (zopiclone and zolpidem). Each patient with general anesthesia was randomly matched to a patient with neuraxial anesthesia, using a frequency-matched-pair procedure [20].

### 2.3. Study Outcome

The primary outcome was NPUSH within 180 days after surgery. The secondary outcomes were NPUSH with a concomitant diagnosis of sleep disorders within 180 days after surgery, NPUSH which occurred 90–180 days after surgery, and NPUSH within 30, 60, 90, 120, and 150 days after surgery. We identified patients who had a postoperative diagnosis of sleep disorder using the International Classification of Diseases, 9th Revision, Clinical Modification (ICD-9-CM) codes [21] (Supplementary Table S1).

### 2.4. Patient and Clinical Characteristics

Insurance premium was classified into $0–$500, $501–$800, and > $800 United States dollars per month. Surgeries were classified into orthopedic (lower extremity), genitourinary, anorectal, obstetric (including cesarean section), and hernia repair surgeries. The ICD-9-CM codes of physicians’ diagnoses within 24 months prior to surgery were used to ascertain the following coexisting diseases, chosen based on data availability, physiological plausibility, and the existing literature: hypertension, diabetes mellitus, ischemic heart disease, atherosclerosis, heart failure, cerebrovascular disease, chronic kidney disease, chronic obstruction pulmonary disease, malignancy, anxiety disorder, depressive disorder, schizophrenia, and bipolar disorder [22] (Supplementary Table S1). Lifestyle factors included obesity, smoking disorder, alcohol-use disorder, other substance-use disorder, and malnutrition [22]. The numbers of hospitalizations and emergency visits within 24 months before the index surgery were examined to reflect patients’ overall health and to avoid ascertainment bias. We also evaluated the requirements for blood transfusion (red blood cells, fresh frozen plasma, or platelets) [23,24] and intensive care during the index surgical admission. Major complications that occurred within 30 days after the index surgery were analyzed, including pneumonia, septicemia, acute renal failure, pulmonary embolism, deep vein thrombosis, stroke, urinary tract infection, surgical site infection, acute myocardial infarction, cardiac dysrhythmias, and postoperative bleeding. The analyses also adjusted for the concurrent medications prescribed within 180 days after the surgery which might cause sleep disorders, including systemic steroids, ephedrine, theophylline, diuretics, and anti-depressants [25]. Diuretics included furosemide, bumetanide, torsemide, spironolactone, and chlorothiazide. Anti-depressants were comprised of selective serotonin reuptake inhibitors (fluoxetine, paroxetine, sertraline, fluvoxamine, and escitalopram) and serotonin norepinephrine reuptake inhibitors (venlafaxine and duloxetine).

### 2.5. Statistical Analysis

Continuous variables were summarized using mean ± standard deviation. Categorical variables were expressed as frequency and percentage. A non-parsimonious multivariable logistic regression model was used to estimate a propensity score for subjects undergoing general or neuraxial anesthesia. We matched each patient with general anesthesia to a patient with neuraxial anesthesia using a greedy matching algorithm within a tolerance limit of 0.05 and without replacement to balance the distributions of age, sex, insurance premium, types of surgery, coexisting diseases, lifestyle factors, concurrent medications, numbers of hospitalizations and emergency visits before surgery, postoperative complications, perioperative blood transfusions, and admission to intensive care units (ICU) between the two groups [20]. The distributions of baseline patient characteristics were compared between matched pairs by using the standardized difference [26]. Multivariable logistic regression analyses were used to adjust for all included variables and to calculate the adjusted odds ratio (aOR) and 95% confidence interval (CI) for the outcome of interest. Kaplan–Meier curves and log-rank tests were used to compare the cumulative incidence of NPUSH within 180 days after surgery between the groups. Subgroup analyses were also conducted by age, sex, coexisting diseases, concurrent medications, postoperative complications, blood transfusions, and admission to the ICU. Sensitivity analyses were conducted by excluding patients who had a history of anxiety disorder, depressive disorder, schizophrenia, bipolar disorder, alcohol-use disorder, other substance-use disorder, or uses of anti-depressants (Analysis I), excluding patients with a history of malignancy (Analysis II), and excluding patients with perioperative uses of blood transfusion, postoperative complications, or ICU admission (Analysis III). We considered a two-sided level of 0.05 statistically significant. All the statistical analyses were conducted using Statistics Analysis System (SAS), Version 9.4 (SAS Institute Inc., Cary, NC, USA).

## 3. Results

### 3.1. Baseline Patient Characteristics

**Figure 1:**
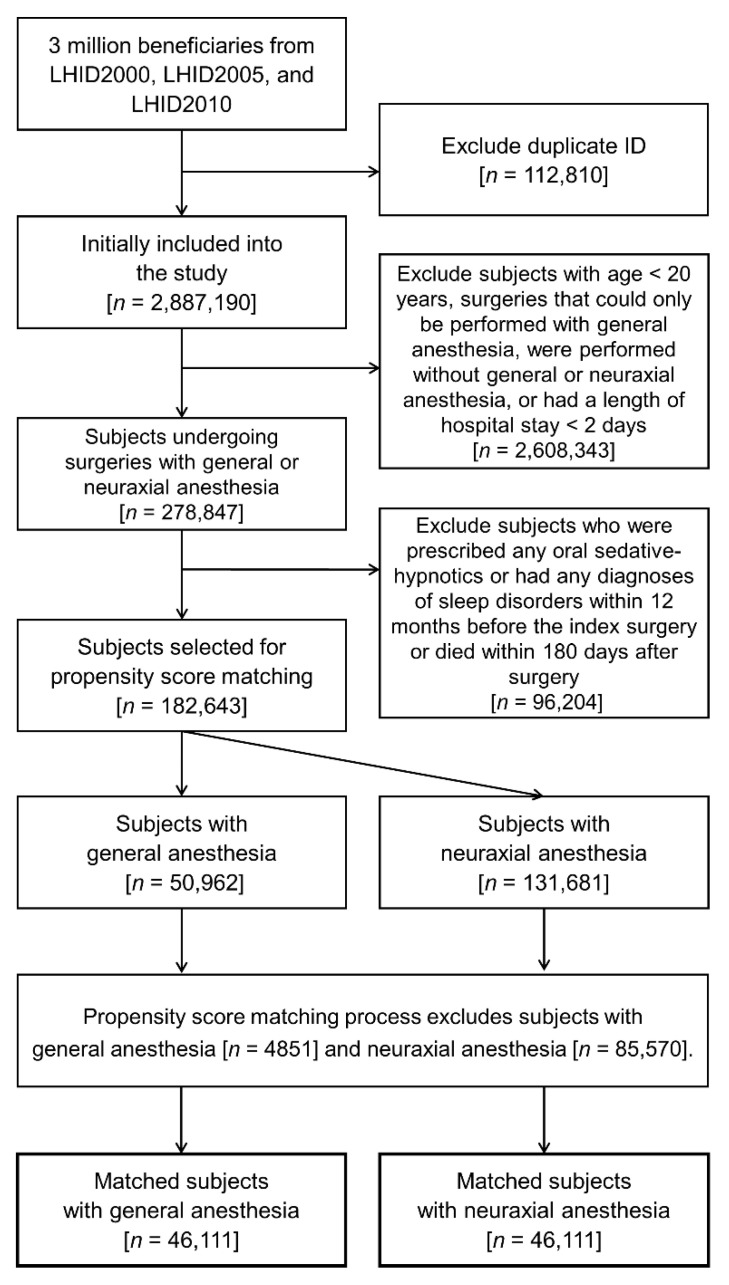
Flow diagram for patient selection.

The patient selection and matching process generated 46,111 matched pairs for analysis. (Figure 1) Table 1 shows the baseline patient characteristics. Notably, the distributions of demographics, types of surgery, coexisting diseases, lifestyle factors, concurrent medications, number of hospitalizations, number of emergency room visits, postoperative complications, perioperative blood transfusions, and admissions to ICUs were well balanced after matching.

### 3.2. New Postoperative Uses of Sedative–Hypnotics

**Figure 2:**
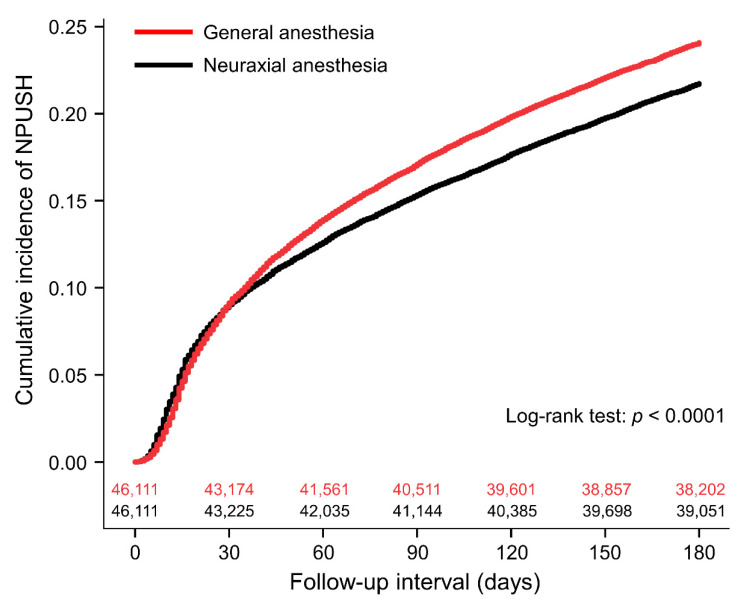
Cumulative incidence of new postoperative uses of sedative–hypnotics (NPUSH) between patients undergoing general and neuraxial anesthesia with number of subjects at risk.

In the postoperative 180-day period, 15,016 patients (16.3%) had NPUSH and 2183 (4.7%) had a concomitant diagnosis of sleep disorders. Table 2 shows the results of univariate and multivariable logistic regression analyses for NPUSH. General anesthesia was significantly associated with a higher risk of NPUSH compared with neuraxial anesthesia (aOR: 1.17, 95% CI: 1.13–1.22, *p* < 0.0001; absolute risk difference: 0.024, 95% CI: 0.017–0.030; Figure 2). The time to NPUSH was median 47 days (interquartile range: 19–100) for patients with general anesthesia and 44 (16–103) for those with neuraxial anesthesia. Sensitivity analyses showed similar results: Analysis I (aOR: 1.18, 95% CI: 1.14–1.23, *p* < 0.0001), Analysis II (aOR: 1.17, 95% CI: 1.12–1.21, *p* < 0.0001), and Analysis III (aOR: 1.18, 95% CI: 1.13–1.22, *p* < 0.0001). In addition, general anesthesia was associated with increased NPUSH with sleep disorders (aOR: 1.11, 95% CI: 1.02–1.21, *p* = 0.0212). General anesthesia was also linked to NPUSH which occurred 90–180 days after surgery (aOR: 1.12, 95% CI: 1.06–1.19, *p* = 0.0002) (Table 3).

Other independent factors for NPUSH were age (aOR: 1.01), sex (male vs. female, aOR: 0.80), insurance premium ($501–800 USD/month vs. 0–500: aOR: 0.95; ≥ 801 vs. 0–500, aOR: 0.79), orthopedic surgery (aOR: 1.44), and obstetric surgery (aOR: 0.50). Coexisting diseases related to NPUSH were ischemic heart disease (aOR: 1.15), heart failure (aOR: 0.86), chronic obstructive pulmonary disease (aOR: 1.10), malignancy (aOR: 1.22), anxiety disorder (aOR: 1.46), schizophrenia (aOR: 1.85), alcohol-use disorder (aOR: 1.75), and other substance-use disorder (aOR: 4.95). Patients using the following medications had a higher risk of NPUSH: systemic steroids (aOR: 1.81), ephedrine (aOR: 1.31), theophylline (aOR: 1.27), diuretics (aOR: 1.88), and anti-depressants (aOR: 16.09). In addition, perioperative blood transfusion (aOR: 2.06), postoperative complications (aOR: 1.29), and ICU admission (aOR: 1.93) were significantly associated with NPUSH. (Table 2)

### 3.3. Subgroup Analyses

General anesthesia was associated with NPUSH compared with neuraxial anesthesia in the subgroups of age < 65 years (aOR: 1.25), no malignancy history (aOR: 1.17), no preoperative anxiety disorder (aOR: 1.19), no use of ephedrine (aOR: 1.21), no use of anti-depressants (aOR: 1.17), no perioperative use of blood transfusion (aOR: 1.17), and no admission to an ICU (aOR: 1.18) (Table 4).

## 4. Discussion

The present study demonstrated that general anesthesia was associated with greater NPUSH compared with neuraxial anesthesia. The NPUSH risk associated with general anesthesia persisted 90 to 180 days after surgery. Our analyses identified some potentially modifiable factors for NPUSH, which may contribute to risk stratification and prevention before surgery. This study has several strengths to evaluate the putative effect of general anesthesia on NPUSH. First, we used a nationwide dataset to increase the patient sample and to cover the medical institutions of different levels, which increases the generalizability of the study results. Second, we used a propensity-score-matching analysis to balance the distributions of various patient and clinical factors and to minimize potential confounding effects. Our results suggest that types of anesthesia may impact the risk of new prescriptions of sedative–hypnotics among surgical patients, providing an implication in preventing the long-term misuse of these drugs.

This study is the first to compare the risk of NPUSH between general and neuraxial anesthesia among surgical patients. Most of the previous studies focused on polysomnography parameters instead of pragmatic outcomes (e.g., sedative–hypnotic prescriptions) [6,7,8,9,11,12,13]. In addition, prior studies did not evaluate the potential impact of different anesthesia techniques on sleep disturbances and sedative–hypnotic uses [6,8,9,10,11,12,13]. Our results suggested that patients receiving neuraxial anesthesia did have NPUSH and sleep disorders, but the risk was significantly lower than that of general anesthesia. Previous studies reported several risk factors for postoperative sleep disorders, including older age [8,27], more extensive surgical trauma [28], and longer length of hospital stay [10]. A recent study showed that perioperative benzodiazepine use was associated with postoperative persistent benzodiazepine use [14]. Our study added important evidence to the current literature by identifying more risk factors for NPUSH, including orthopedic surgery, preexisting malignancy and anxiety disorder, concurrent uses of systemic steroids, ephedrine, theophylline, diuretics, and anti-depressants, perioperative blood transfusion, postoperative complications, and admission to ICUs.

We proposed the following possible explanations for our findings. First, opioids and volatile anesthetics used in general anesthesia may disrupt the sleep/wake cycle and other circadian rhythms (e.g., melatonin secretion and body temperature) [5,6,7,8,9], although it remains controversial whether neuraxial anesthesia effectively reduces the postoperative uses of opioids [29]. Song and colleagues recently showed that subarachnoid anesthesia was related to less impairment of melatonin circadian rhythms and sleep patterns among elderly patients undergoing hip-fracture surgery [30]. Second, pain intensity is an established determinant for postoperative sleep quality, and vice versa [31,32]. Regional anesthesia has proven effective in reducing postoperative acute and chronic pain [33,34]. Third, surgery requiring general anesthesia might reflect the longer operative duration and more extensive surgical injury, which were potentially related to the complicated postoperative course and sleep deprivation. Noticeably, our results have been controlled for postoperative complications and the need for intensive care in the analytical model.

A database study showed that 15.2% and 4.9% of patients with new benzodiazepine prescriptions continued to use benzodiazepines for 1 year and 8 years, respectively [35]. Additionally, postoperative sleep deprivation is associated with delirium, higher sensitivity to pain, and longer length of hospital stay [10,31,36]. However, there are still few prophylactic and therapeutic measures to reduce postoperative sedative–hypnotic uses and to improve postoperative sleep quality. Avoiding perioperative benzodiazepine use may prevent persistent benzodiazepine use after surgery [14]. Furthermore, some clinical strategies have been developed to improve postoperative sleep, including laparoscopic techniques [37], melatonin supplementation [38], and dexmedetomidine infusion [39]. A clinical trial recently reported that propofol-based general anesthesia might promote postoperative sleep quality compared with volatile general anesthesia [40]. Our results indicated that regional anesthesia might protect surgical patients against postoperative sedative–hypnotic uses and sleep disorders, although the effect size appeared modest after adjustment for covariates.

The present study identified several modifiable risk factors for NPUSH. First, systemic corticosteroids, ephedrine, and diuretics are commonly used in the perioperative period. More studies are needed to evaluate their potential impact and threshold dose for postoperative sleep disturbances and NPUSH. Second, perioperative allogeneic blood transfusion has been found to trigger systemic inflammation and potentially exert a detrimental effect on postoperative outcome [41]. In addition, the need for blood transfusion might reflect the longer duration of surgery and greater extent of surgical trauma. It is important to take the risks of NPUSH and sleep disorders into account when blood transfusion is considered for surgical patients. Third, sleep disturbance is common in patients admitted to ICU and is linked to functional disability after critical illness [42,43]. For patients at high risk of NPUSH, sleep medicine or psychiatric consultations may be required to improve postoperative sleep and to prevent sedative–hypnotic misuse. Future studies are warranted to evaluate the potential effect of modifiable disruptors to patient sleep in ICUs (e.g., noise, light, and patient care activities) on the long-term risk of sedative–hypnotic dependence and misuse [42].

There are some limitations to our study. First, our data did not contain information about objective physical measures (e.g., polysomnography parameters), biochemical laboratory tests (e.g., inflammation markers), the American Society of Anesthesiologists physical status, pain intensity, and clinical data on detailed surgical (e.g., elective, emergency, or urgent surgery, wound size, and operative duration) and anesthetic management (e.g., types and doses of opioids and non-opioid anesthetic drugs) that were not covered by the NHI research database. Second, it is possible that anesthesiologists may have chosen to prescribe general anesthesia to patients with an undocumented and untreated history of general anxiety disorders or other borderline psychiatric conditions [44,45]. The psychological predisposition and undiagnosed anxiety disorder could not be adjusted for in the multivariable analyses due to the lack of relevant data. In addition, although we did not consider the use of anxiolytics in the patient selection, the included benzodiazepine drugs are commonly used as preoperative anxiolytics [46]. Third, the indications for postoperative sedative–hypnotic prescriptions were unknown in some patients. Therefore, the biological mechanism of anesthesia-related NPUSH remains to be investigated. Fourth, we did not evaluate the sedative–hypnotic use beyond 180 days after surgery. It is uncertain whether the NPUSH developed into a long-term dependence or misuse. Fifth, this study did not include patients receiving peripheral nerve blocks due to its small patient sample and analytical difficulty in matching three groups. Last, our cohort was only followed up until December 31, 2013, due to the regulations of the NHI research database.

## 5. Conclusions

Patients undergoing general anesthesia had an increased risk of NPUSH and sleep disorders compared with neuraxial anesthesia among surgical patients. The general-anesthesia-related NPUSH risk persisted 90 to 180 days after surgery. More studies are needed to clarify the potential causal relationship and biological mechanism, and to evaluate the potential impact on anesthesia care.

## Figures and Tables

**Table 1:** Baseline characteristics of patients undergoing general and neuraxial anesthesia.

	GA *n* = 46,111		NA *n* = 46,111		SDD
Age (years), mean (SD)	50.0	18.2	50.0	18.7	<0.0001
Sex, male, *n* (%)	25,607	55.5	25,544	55.4	0.0030
Insurance premium (USD/month), *n* (%)					−0.0004
0–500	18,408	39.9	18,477	40.1	
501–800	15,470	33.6	15,319	33.2	
≥801	12,233	26.5	12,315	26.7	
Type of surgery, *n* (%)					
Orthopedic, lower extremity	18,915	41.0	18,957	41.1	−0.0021
Genitourinary	11,906	25.8	11,821	25.6	0.0053
Anorectal	5545	12.0	5434	11.8	0.0127
Obstetric	5968	12.9	5957	12.9	0.0012
Hernia repair	3924	8.5	4108	8.9	−0.0277
Coexisting diseases, *n* (%)					
Hypertension	11,001	23.9	11,013	23.9	−0.0008
Diabetes mellitus	5304	11.5	5341	11.6	−0.0043
Ischemic heart disease	3248	7.0	3280	7.1	−0.0058
Atherosclerosis	272	0.6	261	0.6	0.0229
Heart failure	948	2.1	943	2.1	0.0030
Cerebrovascular disease	2482	5.4	2460	5.3	0.0052
Chronic kidney disease	1592	3.5	1551	3.4	0.0149
COPD	2829	6.1	2889	6.3	−0.0123
Malignancy	2349	5.1	2436	5.3	−0.0211
Anxiety disorder	1725	3.7	1716	3.7	0.0030
Depressive disorder	93	0.2	100	0.2	−0.0401
Schizophrenia	80	0.2	77	0.2	0.0211
Bipolar disorder	30	0.1	33	0.1	−0.0526
Lifestyle factors, *n* (%)					
Obesity	230	0.5	228	0.5	0.0048
Smoking disorder	278	0.6	279	0.6	−0.0020
Alcohol-use disorder	414	0.9	409	0.9	0.0068
Other substance-use disorder	8	0.02	8	0.02	0
Malnutrition	241	0.5	230	0.5	0.0259
Concurrent medications, *n* (%)					
Systemic steroids	6725	14.6	6703	14.5	0.0021
Ephedrine	7302	15.8	7444	16.1	−0.0126
Theophylline	3753	8.1	3827	8.3	−0.0117
Diuretics	3867	8.4	3883	8.4	−0.0025
Anti-depressants	356	0.8	360	0.8	−0.0062
Number of hospitalizations, *n* (%)					0.0090
0	37,741	81.9	37,926	82.3	
1	6085	13.2	5971	13.0	
2	1492	3.2	1412	3.1	
≥3	793	1.7	802	1.7	
Number of ER visits, *n* (%)					−0.0096
0	28,625	62.1	28,500	61.8	
1	10,797	23.4	10,769	23.4	
2	3855	8.4	3892	8.4	
≥3	2834	6.2	2950	6.4	
Blood transfusion, *n* (%)	560	1.2	487	1.1	0.0779
Postoperative complications, *n* (%)	5126	11.1	5422	11.8	−0.0349
ICU admission, *n* (%)	276	0.6	251	0.5	0.0526

Abbreviation: COPD = chronic obstruction pulmonary disease; ER = emergency room; GA = general anesthesia; ICU = intensive care unit; NA = neuraxial anesthesia; SD = standard deviation; SDD = standardized difference; USD = United States dollar.

**Table 2:** Univariate and multivariable analyses for new postoperative uses of sedative–hypnotics.

	Univariate			Multivariable		
	cOR	95% CI	*p*	aOR	95% CI	*p*
GA vs. NA	1.15	1.11–1.19	<0.0001	1.17	1.13–1.22	<0.0001
Age (years)	1.03	1.02–1.03	<0.0001	1.01	1.01–1.01	<0.0001
Sex, male vs. female	0.85	0.83–0.89	<0.0001	0.80	0.76–0.83	<0.0001
Insurance premium (USD/month)			<0.0001			<0.0001
501–800 vs. 0–500	0.81	0.78–0.84	<0.0001	0.95	0.91–0.99	0.0008
≥801 vs. 0–500	0.50	0.47–0.52	<0.0001	0.79	0.74–0.83	<0.0001
Type of surgery						
Orthopedic, lower extremity	1.79	1.73–1.85	<0.0001	1.44	1.04–2.01	0.0303
Genitourinary	1.04	1.00–1.08	0.0823	1.04	0.75–1.45	0.7960
Anorectal	0.75	0.70–0.79	<0.0001	1.08	0.78–1.51	0.6460
Obstetric	0.34	0.31–0.36	<0.0001	0.50	0.36–0.71	<0.0001
Hernia repair	0.64	0.59–0.69	<0.0001	0.79	0.57–1.10	0.1610
Coexisting diseases						
Hypertension	1.81	1.74–1.88	<0.0001	0.96	0.92–1.01	0.1280
Diabetes mellitus	1.67	1.59–1.75	<0.0001	1.06	1.00–1.12	0.0555
Ischemic heart disease	1.91	1.80–2.02	<0.0001	1.15	1.07–1.23	<0.0001
Atherosclerosis	2.16	1.79–2.60	<0.0001	1.18	0.96–1.44	0.1142
Heart failure	2.02	1.82–2.23	<0.0001	0.86	0.77–0.97	0.0122
Cerebrovascular disease	1.77	1.66–1.90	<0.0001	0.93	0.86–1.00	0.0608
Chronic kidney disease	1.64	1.51–1.79	<0.0001	0.95	0.86–1.04	0.2863
COPD	1.82	1.71–1.93	<0.0001	1.10	1.02–1.18	0.0106
Malignancy	1.62	1.51–1.74	<0.0001	1.22	1.13–1.31	<0.0001
Anxiety disorder	1.82	1.68–1.97	<0.0001	1.46	1.34–1.59	<0.0001
Depressive disorder	1.85	1.34–2.55	0.0002	1.02	0.70–1.47	0.9374
Schizophrenia	2.41	1.72–3.37	<0.0001	1.85	1.28–2.67	0.0010
Bipolar disorder	3.17	1.90–5.27	<0.0001	1.76	0.98–3.17	0.0586
Lifestyle factors						
Obesity	1.29	1.03–1.63	0.0274	1.16	0.91–1.48	0.2230
Smoking disorder	1.00	0.80–1.26	0.9718	1.12	0.89–1.41	0.3489
Alcohol-use disorder	1.82	1.56–2.13	<0.0001	1.75	1.48–2.06	<0.0001
Other substance-use disorder	5.15	1.93–13.71	0.0011	4.95	1.78–13.72	0.0021
Malnutrition	1.44	1.16–1.80	0.0010	0.93	0.73–1.18	0.5417
Concurrent medications						
Systemic steroids	2.32	2.22–2.42	<0.0001	1.81	1.73–1.89	<0.0001
Ephedrine	1.27	1.21–1.33	<0.0001	1.31	1.24–1.37	<0.0001
Theophylline	1.78	1.69–1.89	<0.0001	1.27	1.20–1.36	<0.0001
Diuretics	3.13	2.97–3.29	<0.0001	1.88	1.77–1.99	<0.0001
Anti-depressants	16.97	14.28–20.17	<0.0001	16.094	13.42–19.30	<0.0001
Number of hospitalizations			<0.0001			0.7140
1 vs. 0	1.24	1.18–1.31	0.0018	1.01	0.95–1.06	0.3104
2 vs. 0	1.45	1.32–1.58	0.0577	0.98	0.88–1.08	0.9860
≥3 vs. 0	1.83	1.63–2.05	<0.0001	0.93	0.81–1.07	0.3301
Number of ER visits			<0.0001			0.1101
1 vs. 0	1.02	0.98–1.06	0.0003	0.98	0.93–1.02	0.4005
2 vs. 0	1.02	0.96–1.09	0.0133	0.95	0.88–1.01	0.0635
≥3 vs. 0	1.35	1.26–1.44	<0.0001	1.06	0.98–1.14	0.0433
Blood transfusion	4.79	4.24–5.42	<0.0001	2.06	1.78–2.39	<0.0001
Postoperative complications	1.68	1.60–1.77	<0.0001	1.29	1.22–1.36	<0.0001
ICU admission	4.39	3.69–5.21	<0.0001	1.93	1.55–2.42	<0.0001

Abbreviation: aOR = adjusted odds ratio; COPD = chronic obstruction pulmonary disease; cOR = crude odds ratio; ER = emergency room; GA = general anesthesia; ICU = intensive care unit; NA = neuraxial anesthesia; USD = United States dollar.

**Table 3:** New postoperative uses of sedative–hypnotics for patients undergoing general or neuraxial anesthesia.

	GA		NA		NPUSH risk	
	Event	Rate (%)	Event	Rate (%)	aOR (95% CI) ^†^	*p*
All NPUSH	7938	17.2	7078	15.4	1.17 (1.13–1.22)	<0.0001
NPUSH with sleep disorder	1135	2.5	1048	2.3	1.11 (1.02–1.21)	0.0212
30-day NPUSH	3011	6.5	2943	6.4	1.03 (0.98–1.09)	0.2527
60-day NPUSH	4587	10.0	4107	8.9	1.15 (1.10–1.20)	<0.0001
90-day NPUSH	5640	12.2	4994	10.8	1.17 (1.12–1.22)	<0.0001
120-day NPUSH	6539	14.2	5760	12.5	1.18 (1.14–1.23)	<0.0001
150-day NPUSH	7279	15.8	6436	14.0	1.18 (1.14–1.23)	<0.0001
90–180-day NPUSH	2338	5.1	2111	4.6	1.12 (1.06–1.19)	0.0002

Abbreviation: aOR = adjusted odds ratio; CI = confidence interval; GA = general anesthesia; NA = neuraxial anesthesia; NPUSH = new postoperative uses of sedative–hypnotics. ^†^ Adjusted for age (continuous), sex, insurance premium (categorical), types of surgery, coexisting diseases, lifestyle factors, concurrent medications, number of hospitalizations, number of emergency room visits, perioperative uses of blood transfusion, postoperative complications, and intensive care unit care.

**Table 4:** Subgroup analyses of new postoperative uses of sedative–hypnotics for patients undergoing general or neuraxial anesthesia.

Subgroup		*n*	Event	Rate (%)	aOR (95% CI) ^†^	*p*
Age ≥ 65 years	GA	11,147	2789	25.0	1.03 (0.97–1.10)	0.3518
	NA	11,921	2941	24.7	reference	
Age < 65 years	GA	34,964	5149	14.7	1.25 (1.20–1.31)	<0.0001
	NA	34,190	4137	12.1	reference	
Male	GA	25,607	4143	16.2	1.13 (1.08–1.19)	<0.0001
	NA	25,544	3695	14.5	reference	
Female	GA	20,504	3795	18.5	1.20 (1.14–1.27)	<0.0001
	NA	20,567	3383	16.5	reference	
Malignancy history	GA	2349	568	24.2	1.15 (1.00–1.33)	0.0566
	NA	2436	554	22.7	reference	
No malignancy history	GA	43,762	7370	16.8	1.17 (1.12–1.21)	<0.0001
	NA	43,675	6524	14.9	reference	
Anxiety disorder	GA	1725	421	24.4	0.90 (0.77–1.06)	0.1962
	NA	1716	460	26.8	reference	
No anxiety disorder	GA	44,386	7517	16.9	1.19 (1.14–1.23)	<0.0001
	NA	44,395	6618	14.9	reference	
Use of systemic steroids	GA	6725	1964	29.2	1.16 (1.07–1.26)	0.0003
	NA	6703	1784	26.6	reference	
No use of systemic steroids	GA	39,386	5974	15.2	1.17 (1.12–1.22)	<0.0001
	NA	39,408	5294	13.4	reference	
Use of ephedrine	GA	7302	1425	19.5	1.02 (0.93–1.11)	0.6935
	NA	7444	1399	18.8	reference	
No use of ephedrine	GA	38,809	6513	16.8	1.21 (1.16–1.26)	<0.0001
	NA	38,667	5679	14.7	reference	
Use of theophylline	GA	3753	954	25.4	1.12 (1.01–1.25)	0.0387
	NA	3827	918	24.0	reference	
No use of theophylline	GA	42,358	6984	16.5	1.18 (1.13–1.22)	<0.0001
	NA	42,284	6160	14.6	reference	
Use of diuretics	GA	3867	1400	36.2	1.13 (1.03–1.25)	0.0116
	NA	3883	1298	33.4	reference	
No use of diuretics	GA	42,244	6538	15.5	1.18 (1.13–1.22)	<0.0001
	NA	42,228	5780	13.7	reference	
Use of anti-depressants	GA	356	283	79.5	1.18 (0.79–1.75)	0.4150
	NA	360	262	72.8	reference	
No use of anti-depressants	GA	45,755	7655	16.7	1.17 (1.13–1.21)	<0.0001
	NA	45,751	6816	14.9	reference	
Postoperative complications	GA	5126	1271	24.8	1.17 (1.07–1.29)	0.0011
	NA	5422	1196	22.1	reference	
No postoperative complication	GA	40,985	6667	16.3	1.17 (1.13–1.22)	<0.0001
	NA	40,689	5882	14.5	reference	
Blood transfusion	GA	560	272	48.6	1.09 (0.84–1.43)	0.5240
	NA	487	226	46.4	reference	
No blood transfusion	GA	45,551	7666	16.8	1.17 (1.13–1.22)	<0.0001
	NA	45,624	6852	15.0	reference	
ICU admission	GA	276	124	44.9	0.77 (0.50–1.20)	0.2494
	NA	251	117	46.6	reference	
No ICU admission	GA	45,835	7814	17.1	1.18 (1.13–1.22)	<0.0001
	NA	45,860	6961	15.2	reference	

Abbreviation: aOR = adjusted odds ratio; CI = confidence interval; GA = general anesthesia; ICU = intensive care unit; NA = neuraxial anesthesia. ^†^ Adjusted for age (continuous), sex, insurance premium (categorical), types of surgery, coexisting diseases, lifestyle factors, concurrent medications, number of hospitalizations, number of emergency room visits, perioperative uses of blood transfusion, postoperative complications, and intensive care unit care.

## Data Availability

The data presented in this study are available on request from the corresponding author. The data are not publicly available due to the regulations of the Institutional Review Board.

## Supplementary Materials

The following supporting information can be downloaded at: https://www.mdpi.com/article/10.3390/jcm11123360/s1, Table S1. ICD-9-CM codes of coexisting diseases, lifestyle factors, postoperative complications, and outcomes.
