# Supplementary material for: The Association between General Anesthesia and New Postoperative Uses of Sedative–Hypnotics: A Nationwide Matched Cohort Study
Source: J Clin Med. 2022 Jun 11;11(12):3360. doi: 10.3390/jcm11123360 (PMC9224548; doi:10.3390/jcm11123360)
Supplement: Supplementary file 1 [file jcm-11-03360-s001.zip › Supplementary table.pdf]

Table S1. ICD-9-CM codes of coexisting diseases, lifestyle factors, postoperative complications, and outcomes

**Coexisting disease**	
Hypertension	401-405
Diabetes mellitus	250
Ischemic heart disease	410-414
Atherosclerosis	440
Heart failure	428
Cerebrovascular disease	430-438
Chronic kidney disease	585
Chronic obstructive pulmonary disease	490, 491, 496
Malignancy	140-208, 230-234
Anxiety disorder	300
Depressive disorder	296.2, 296.3
Schizophrenia	295
Bipolar disorder	296, except 296.2x, 296.3x, 296.9x, 296.82
**Lifestyle factor**	
Obesity	278
Smoking disorder	V15.82, 305.1
Alcohol use disorder	291.0, 291.1, 291.2, 291.3, 291.4, 291.5, 291.8, 291.81, 291.82, 291.89, 291.9, 303.00-303.03, 303.90-303.93, 305.00-305.03, 357.5, 425.5, 535.30, 535.31, 571.0, 571.1, 571.2, 571.3, E860.0
Other substance use disorders	304.40-304.43, 305.70-305.73, 304.30-304.33, 305.20-305.23, 304.20-304.23, 305.60-305.63, 968.5, E938.5, 304.50-304.53, 305.30-305.33, 969.6, E854.1, E939.6, 304.00-304.03, 304.70-304.73, 305.50-305.53, 965.00, 965.01, 965.02, 965.09, E850.0, E935.0, 304.10-304.13, 305.40-305.43
Malnutrition	262, 263.0, 263.1, 263.8, 263.9, 783.22, 783.21, 799.4, V85.0, 260, 261
**Postoperative complication**	
Pneumonia	480-486
Septicemia	038
Acute renal failure	584
Pulmonary embolism	415.1
Deep vein thrombosis	451.11, 451.19, 451.2, 451.81, 451.9, 453.40-453.42, 453.8, 453.9
Stroke	430-437
Urinary tract infection	599.0
Surgical site infection	682, 682.6, 682.9, 686.8, 686.9, 998.5, 998.51, 998.59
Acute myocardial infarction	410
Cardiac dysrhythmias	427
Postoperative bleeding	998.1
**Outcome**	
Sleep disorder	307.41, 307.42, 780.52, 327.2, 780.51, 780.53, 780.57
